# Genetic dissection of gluten characteristics based on single- and multi-locus genome-wide association studies in wheat (*Triticum aestivum* L.)

**DOI:** 10.1016/j.fochms.2025.100342

**Published:** 2025-12-13

**Authors:** Xiaoling Jiang, Qiang Li, Yanyan Geng, Jishun Zhao, Yang Li, Hongmin Li

**Affiliations:** a College of Agriculture and Forestry Science, Hebei North University, Zhangjiakou 075000, Hebei, PR China; b Hebei Key Laboratory of Quality & Safety Analysis-Testing for Agro-Products and Food, Hebei North University, PR China; c Dryland Farming Institute, Hebei Academy of Agriculture and Forestry Sciences, Hengshui 053000, China; d Hebei University of Engineering, Handan 056038, Hebei, PR China

**Keywords:** Gluten, Multi-locus genome-wide association study, Single-locus genome-wide association study, Candidate gene, Network analysis, KASP markers

## Abstract

Gluten protein quantity and quality, crucial factors determining the baking quality of wheat-based foods, are primary targets for wheat breeding. To elucidate their genetic basis, five key gluten traits were investigated utilizing a genome-wide association study (GWAS) approach: wet gluten content (WGC), residual gluten content (RGC), dry gluten content (DGC), water-holding capacity (WHC), and gluten index (GI). Using 48,057 SNPs across 200 wheat accessions, analyses employed one single-locus (SL) model and five multi-locus (ML) models. Genotype primarily influenced these gluten traits, with broad-sense heritability (H^2^) ranging from 0.85 (DGC) to 0.97 (GI). The SL-GWAS and ML-GWAS models identified 143 and 203 significant marker-trait associations (MTAs), respectively. Of these, 15 stable quantitative trait loci (QTL) were detected in at least three environments using multiple GWAS models. Most notably, qGI·1D for GI, which integrated from 138 significant MTAs, was identified in multi-environments and recognized by all five ML-GWAS models across all environments. This QTL was shown to be co-localized with qWGC·1D, qRGC·1D, and qWHC·1D. Furthermore, five candidate genes related to wheat gluten including *TraesCS1A02G317500*, *TraesCS1A02G466400LC*, *TraesCS1A02G466500LC*, *TraesCS1B02G330000*, and *TraesCS1D02G317300* were indentified. Interestingly, *TraesCS1B02G330000* has the PF13016 domain related to gliadins and has collinearity with two other genes, suggesting the genes in the first homologous group encoding gliadins may play an important role in GI. Additionally, four kompetitive allele-specific PCR (KASP) markers (*K_AX-108*,*999*,*948*, *K_AX-110*,*940*,*435*, *K_AX-111*,*216*,*618* and *K_AX-94*,*670*,*671*) for GI were developed successfully and validated in the natural population. This work elucidates the genetic basis of wheat gluten traits and provides both valuable germplasm and robust molecular tools for breeding applications.

## Highlights

• Wheat gluten plays a crucial role in determining the baking quality of wheat-based foods.
• Due to the time-consuming and costly nature of obtaining phenotype data for gluten traits, there has been limited progress in related research
• Identified 15 QTLs with gluten-related traits, including a major locus (*qGI.1D*).
• Four KASP markers for GI were developed.

## 1 Introduction

Common wheat (*Triticum aestivum* L.), a major cereal crop cultivated worldwide, supplies around 20 % of total caloric intake and 23 % of dietary protein for human consumption (Gao et al., 2021; Shewry & Hey, 2015). Wheat flour contains a variety of components, including carbohydrates, protein, lipids, and enzymes. Proteins, which constitute the primary component of the wheat endosperm, exert a profound influence on the processing attributes of wheat (Zhou et al., 2021). Gluten, accounting for 80–85 % of the total protein content in wheat flour, is instrumental in determining the elasticity, cohesiveness, and viscosity of dough(He et al., 2020; Lagrain et al., 2008; Sun et al., 2021). Based on solubility, gluten proteins are classified into gliadins and glutenins. Gliadins impart adhesiveness and extensibility to the dough, whereas glutenins confer strength and elastic properties. (Gianibelli et al., 2001; Labuschagne et al., 2021; Shewry & Hey, 2015; Xu et al., 2007). Research has demonstrated that the concentration and properties of gluten proteins substantially affect the baking performance of wheat-derived products, including bread, noodles, and steamed bread, among others (Chen et al., 2025; Li, Wang, et al., 2019; Torbica et al., 2007; Zang et al., 2022). Different wheat-based food items necessitate specific gluten protein characteristics. Wheat with a high gluten content and strength, for example, is ideal for baked bread, medium levels for Chinese steamed bread and noodles, and low levels for cookies. Therefore, gluten protein traits are pivotal in assessing wheat flour quality and its suitability for various end uses, and have emerged as a central objective in wheat quality breeding programs (Schimke, 2020; Wouters et al., 2016; Zhou et al., 2021). Consequently, elucidating the genetic foundations of these traits is essential to facilitate rapid and substantial improvements in wheat quality through marker-assisted selection (MAS) and related biotechnological approaches.

Gluten protein-related parameters are quantitative traits that are influenced by the agro-environment and regulated by multiple genes with a complicated genetic background (Deng et al., 2015; Hao et al., 2022). To date, numerous quantitative trait loci (QTLs) for wheat gluten-related parameters have been found on almost all 21 chromosomes of various genetic populations and association panels (Barakat et al., 2020; Deng et al., 2015; Liu et al., 2017; Merida-Garcia et al., 2019). For example, Alemu et al. (2021) employed 802 spring bread wheat breeding lines to uncover 11 extremely significant quantitative trait nucleotides (QTNs) for wet gluten content (WGC) on chromosomes 1A, 2A, 2B, 3B, 3D, 4B, 6A, 6D and 7D. Suliman et al. (2020) detected 29 significant single nucleotide polymorphism (SNP) markers associated with WGC on chromosomes 1A, 1B, 2A, 2B, 4A, 5B, 6A, 7A and 7B utilizing 189 spring bread wheat genotypes and a 15 K wheat SNP assay. Deng et al. (2015) discoverd 22 QTLs for gluten-related traits such as WGC, residue gluten content (RGC) and gluten index (GI) on chromosomes 1A, 1B, 1D, 3B, 4B, 5D, 6A and 6B using two distinct recombinant inbred line populations. *QRgc.sdau-1D* for RGC and *QGi.sdau-1D* for GI shared a common interval on chromosome 1D and contributed for 24.81 % and 42.81 % of the phenotypic variance, respectively. Furthermore, Barakat et al. (2020) used two doubled haploid populations to map about 43 major QTLs for dry gluten content (DGC), WGC and GI across all 21 chromosomes, accounting for 9.0–21.0 % of phenotypic variance. Despite the identification of numerous QTLs for gluten-related traits, consistent QTLs have been restricted due to differences in mapping populations, methodologies, and environments, among other factors. Most critically, few of them are available to wheat breeding programs. As a result, further extensive investigations on genetic architecture and identification of associated loci for wheat gluten-related traits should be conducted to enhance the application of MAS in wheat high-quality breeding.

Genome-wide association study (GWAS) has been widely used to investigate the genetic dissection of complex quantitative traits controlled by multiple genes, especially with the emergence of improved genomic sequencing technologies (Wang et al., 2016). Using a larger range of germplasm, GWAS may phenotype several traits with one cycle of genotyping with higher resolution than QTL mapping by bi-parental populations, which can only focus on single traits (Li, Wen, et al., 2019). The single locus mixed linear model (SL-MLM) was historically employed in GWAS due to its good control of false-positive sites (Adhikari et al., 2020; Mu et al., 2020; Zhang et al., 2008). However, due to the overly conservative Bonferroni correction (0.05/me, where me is the number of effective markers) and the strict significance test threshold, SL-MLM typically has difficulty finding important genes with minor effects (Wang et al., 2016; Yang et al., 2020a), and SL-MLM has been reported to not adequately account for large effect loci (Rakitsch et al., 2013; Segura et al., 2012). Several multi-locus (ML) models have been developed to address this shortcoming, including multi-locus random-SNP-effect (mrMLM) (Wang et al., 2016), fast mrMLM (FASTmrMLM) (Tamba & Zhang, 2018), fast multi-locus random-SNP-effect efficient mixed model analysis (FASTmrEMMA) (Wen et al., 2018), polygenic-background-control-based least angle regression plus empirical Bayes (pLARmEB) (Zhang et al., 2017) and integration of Kruskal-Wallis test with empirical Bayes (pKWmEB) (Ren et al., 2018). ML-GWAS models are considered more efficient and accurate for mapping genomic areas than SL-MLM models because to their simultaneous evaluation of all marker effects (Vikas et al., 2022). ML-GWAS models identify minor-effect QTLs and remove the need for multiple testing corrections, which can lead to false negatives (Yang et al., 2020b; Zhang et al., 2019). Multi-locus GWAS has successfully identified novel and important minor-effect QTLs associated with complex traits in wheat, including quality-related traits (Peng et al., 2018; Yang et al., 2020a), yield-related traits (Lin et al., 2021; Malik et al., 2021; Muhammad et al., 2020), disease resistance (Cabral et al., 2023; Zhang et al., 2022) and salinity tolerance (Chaurasia et al., 2020).

KASP has gained prominence as a vital high-throughput genotyping tool, indispensable for genotyping and MAS breeding, and the establishment of genetic identity maps. Furthermore, KASP may be utilized for variety identification owing to its high genetic stability, excellent repeatability, and compatibility with high-throughput automated analysis, offering an advantage over traditional SSR-based methods (Carvalho et al., 2021). To date, KASP can significantly accelerate the efficiency of the breeding process and has been effectively utilized in cereal crops such as wheat, rice, and maize, as well as in vegetable species like tomato, cucumber and grapevine (Wang et al., 2023).

In the present study, using the wheat 55 K iSelect SNP array, both single-locus and multi-locus GWAS were performed to more thoroughly investigate the genetic architecture of wheat gluten-related traits in an association panel of 200 wheat accessions from six different environments. The study's goals were to (1) estimate the heritability of wheat gluten-related traits across multiple environments, (2) detect novel and trustworthy genomic regions by integrating single-locus and multi-locus GWAS models, (3) identify putative candidate genes for gluten-related traits in wheat, and (4) design and verify KASP markers. The findings of this study will benefit in the development of MAS in high-quality wheat breeding programs.

## 2 Materials and methods

### 2.1 Plant material

The association mapping panel (AMP) employed in this study consisted of 200 diverse wheat genotypes, primarily commercial cultivars, landrace variants, and top breeding strains. There were 174 accessions from 17 Chinese provinces, with the remaining 26 foreign materials from nine other countries (Supplementary Table S1). All accessions were planted in Huixian county (Henan Province, China, 116.41°E, 39.91°N) during the winter cropping seasons of 2014–2015, 2016–2017, 2017–2018 and 2018–2019, denoted as E1, E2, E3 and E4, respectively, and Xinxiang county (Henan Province, China, 113.9°E, 35.2°N) during the 2017–2018 and 2018–2019 cropping seasons, labeled as E5 and E6, respectively. Field trials were carried out in each location using a randomized complete block design with two replicates. Each block had three rows, each two meters long and with a row spacing of 25 cm. Field management adhered to recommended local norms, and no serious pests or lodging were recorded during the development period. Finally, once mature, the accessions were collected individually, sun-baked, and stored in a ventilated room.

### 2.2 Phenotypic evaluation

The flour was manufactured using a Laboratory Mill (LRMM8040–3-D, Wuxi Buhler Machinery Manufacturing Co. Ltd., China), with an extraction rate of roughly 67 %. The AACC 38–11 Glutenmatic (2200, Perten Instruments, Sweden) was used to determine gluten-related traits such as wet gluten content (WGC), residual gluten content (RGC), dry gluten content (DGC), water-holding capacity (WHC), and gluten index (GI).

### 2.3 Statistical analysis and visualization

To exclude environmental effects, the best linear unbiased estimator (BLUE) values for each trait were generated across six different environments (year-location combinations) using the software QTL IciMapping V4.2 (http://www.isbreeding.net), with genotype as a fixed effect in the model, and then analyzed statistically. The five gluten parameters' phenotypic data were statistically evaluated using Microsoft Excel 2016 and the R package lme4 (Bates et al., 2014). The ANOVA function in IciMapping V4.2 (http://www.isbreeding.net/) was used to calculate the analysis of variance (ANOVA) and broad-sense heritability (H^2^). The H^2^ was estimated based on the average values across replications and environments using the following formula: $H^{2}=\frac{V_{G}}{V_{G}+\frac{1}{e}V_{\mathrm{GE}}+\frac{1}{\mathrm{re}}V_{\varepsilon }}$, where VG is the genotypic variance, VGE is the genotype by environment, Vε is the error variance, e is the number of environments, and r is the number of replications. Refer to Jiang, et al. for further information on data computation (Jiang et al., 2023). Sangerbox (Shen et al., 2022), an interactive data analysis platform, was used to create violin plots for each studied parameter in this AMP, an interactive data analysis platform.

### 2.4 Genotyping and quality control

From the young leaf tissues of each wheat accession, total genomic DNA was isolated by employing a modified cetyltrimethylammonium bromide (CTAB) technique (Priyadharshini et al., 2019). Capital Bio Technology Corporation of Beijing, China (http://www.capitalbiotech.com/) genotyped all 200 accessions using the high-density Illumina Infinium iSelect 55 K SNP array. Using the PLINK software (Purcell et al., 2007), 48,057 high-quality SNPs were selected for GWAS in the AMP after filtering out those with MAF ≤0.05, missingness >0.1, and missing data ≥20 %. The subgenomes A, B, and D have 16,407, 17,798, and 13,852 SNPs, covering 4925, 5174, and 3948 million base pairs (Mb) of map length, respectively. The average distance between markers per chromosome ranged from 0.20 Mb (chromosome 1D) to 0.55 Mb (chromosome 4D), with an average marker density of 0.30 Mb (Table S2).

### 2.5 Population structure and linkage disequilibrium analysis

To reduce the impact of SNP bias on population structure estimates, SNPs were filtered using PLINK with three parameters: 1000 window size, 100 step, and 0.2 r2 threshold (Purcell et al., 2007). The population structure was analyzed using 3050 polymorphic SNP markers and STRUCTURE 2.3.4 (Hubisz et al., 2009), a Bayesian model-based clustering tool. Five independent runs were performed for each K value ranging from 1 to 10 cluters to calculate the number of hypothetical subpopulations using an admixture model. Each run included 10,000 burn-in iterations, followed by 100,000 recorded Markov chain Monte Carlo (MCMC) repeats. STRUCTURE HARVESTER was used to infer the most likely number of subgroups using an ad hoc quantity statistic (ΔK) based on the rate of change in log likelihood of data for successive K values (Earl & vonHoldt, 2012). Additionally, the STRUCTURE HARVESTER data were summarized using the tool CLUMPP v1.1.2 (Jakobsson & Rosenberg, 2007). Neighbor-joining (NJ) trees were also used to validate population stratification with Tassel v5.0 software. The relative kinship matrix (K-matrix) between accessions was computed using TASSEL. Finally, the GWAS was performed using the population structure (Q-matrix) of 200 accessions produced by the CLUMPP software, as well as the kinship matrix (K-matrix) between accessions estimated by TASSEL5.2.21 and R package mrMLM, respectively.

Pairwise comparisons of 48, 209 filtered SNP markers utilizing squared allele frequency correlations (r^2^) in TASSEL 5.2.21 were used to assess linkage disequilibrium. LddecayPlotTool (https://aozhangchina.github.io/R/LDdecay/LDdecayPlotTool.html#lddecayplottool) was used to calculate the mean values of r^2^ over scale distance for A, B and D subgenomes separately (Bradbury et al., 2007). The LD decay over physical distance from each subgenomes were visualized with Microsoft Excel 2016. Previous studies defined the QTL confidence interval of each subgenome by setting 0.2 as the cutoff threshold of r^2^. Significant SNPs with a corresponding physical distance at which LD decays at the critical r^2^ value (0.2) were then selected and referred to as a conservative QTL (Khatkar et al., 2008; Yao et al., 2020).

### 2.6 Genome-wide association studies

Five wheat gluten-related traits were investigated simultaneously using a single-locus GWAS model (MLM) and five multi-locus GWAS models.

The single-locus model (SL-MLM) was implemented by software TASSEL 5.2.21 (Bradbury et al., 2007), while the multi-locus model were tested by using mrMLM v4.0 in R (Zhang et al., 2020) with five methods, including mrMLM, FASTmrMLM, FASTmrEMMA, pLARmEB and pKWmEB (Ren et al., 2018; Tamba & Zhang, 2018; Wen et al., 2018). The Bonferroni correction approach was used to identify significant marker-trait associations (MTAs) (Jiang et al., 2023). The criterion for P was established at *P* < 1.04E-06, corresponding to -log10(P) > 5.98, where *P* = 0.05/Meff. Meff is the effective number of SNPs, which was calculated to be 48,057 using the Genetic Type I Error Calculator (GEC) (Li et al., 2012). The R2-value, which is an estimate of the percentage of variance explained by a marker, was used to illustrate its association with a trait.

All significant MTAs within the projected linkage disequilibrium (LD) block coverage were merged into a single QTL region (Pasam et al., 2012). In this study, neighboring significant SNPs with physical distances less than 12.7 Mb were clustered into a single QTL, which was defined based on the least LD attenuation distance for A, B and D subgenomes, and represent SNPs represented the SNPs with stronger phenotypic contribution. Finally, the discovered QTLs were given the following designation: q (denotes “QTL”) + (abbreviation of the linked trait) + (wheat chromosome where the putative QTL was detected). For example, qGI·1D refers to the QTL for GI found on chromosome 1D.

### 2.7 Candidate gene prediction and association network construction

Throughout this study, QTLs that were consistently expressed in various environments or discovered by a minimum of two GWAS models were regarded key and reliable loci for gluten-related traits. The candidate genes were predicted using the highly significant SNPs located in the aforementioned loci, as previously reported (Hu et al., 2020; Jin et al., 2020). In brief, the two-sided SNP markers of these loci were utilized to search for annotated genes in genomic intervals of the Chinese Spring reference genome (IWGSCv1.0, http://www. wheat genome. Org/) using the IntervalTool (Ma et al., 2021). Annotated genes within this interval whose activities were linked to gluten-related phenotypes were deemed to be potential genes. Using NetGWAS (Behrouzi et al., 2017), the association between traits and SNPs that were closely linked to potential genes was further confirmed. The WheatCENet website (Li et al., 2022) was utilized to construct a gene network for candidate genes. Ultimately, these two outcomes were visualized using the Cytoscape program (Shannon et al., 2003).

### 2.8 Establishment and verification of KASP markers

The SNPs consistently identified in different environments were selected, and corresponding KASP markers was successfully converted. Two allele-specifc forward primers and one common reverse primer were designed using the Polymarker (http://polymarker.tgac.ac.uk/). The FAM (5′ GAAGGTGACCAAGTTCATGCT 3′), HEX (5′ GAAGGTCGGAGTCAACGGATT 3′) tails were added to the 5′ end of the two allele-specific primers, respectively, and the sequences of the common primers are listed in Table S5. Each PCR reaction was prepared with a primer mixture comprising 46 μl of ddH_2_O, 12 μl of tailed primer (100 μM), and 30 μl of common primer (100 μM). The PCR protocol consisted of the following steps: an initial hot start at 95 °C for 15 min; 10 touchdown cycles (20 s at 95 °C, 30 s of annealing starting at 65 °C with a decrease of 0.8 °C per cycle); and 10 standard annealing cycles (20 s at 95 °C, 60 s at 57 °C).

## 3 Results

### 3.1 Phenotypic variation of wheat gluten-related traits

**Fig. 1:**
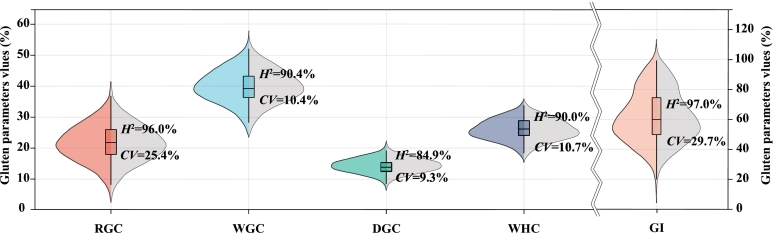
The distribution of five gluten-related traits in the association panel based on BLUE values GI, gluten index; DGC, dry gluten content; WGC, wet gluten content; RGC, residual gluten content; WHC, water-holding capacity; *H*^2^, broad-sense heritability; *CV*, coefficient of variation. (For interpretation of the references to colour in this figure legend, the reader is referred to the web version of this article.)

The distributions of phenotypic data based on BLUE values for all five gluten-related traits in the panel of 200 accessions were shown in violin plots (Fig. 1). The distributions revealed considerable variation as well as the quantitative nature of each parameter. Their coefficient of variation (*CV*) ranged from 9.3 % to 29.7 %, with DGC being the smallest and GI being the largest. All investigated traits demonstrated high broad-sense heritability (*H*^2^), ranging from 85.0 % for DGC to 97.0 % for GI (Fig. 1), implying that genetic factors played a determinant role for each trait. Interestingly, the *CV* and *H*^2^ of WGC, DGC and WHC were comparable and lower, whereas those of RGC and GI were comparable and relatively high. ANOVA indicated that the effects of genotypes, environments, and their interactions (G × E) were highly significant (*P* < 0.0001) for all five gluten-related traits (Table 1). In general, these observed traits demonstrated quantitative nature and robustness, making them suitable for genetic locus identification.

**Table 1:** Analysis of variance for wheat gluten-related traits in the GWAS panel.

Trait	Mean squres				F value		
	Genotype	Environment	G × E	Error	Genotype	Environment	G × E
GI	2742.4	7382.9	84.8	11.7	235.4***	633.6***	7.3***
DGC	9.7	115.6	1.6	0.6	17.5***	208.9***	2.9***
WGC	95.1	746.9	10.0	1.0	92.0***	722.7***	9.6***
RGC	224.3	549.6	9.4	1.9	120.6***	295.4***	5.1***
WHC	38.6	167.5	4.2	1.0	37.9***	164.3***	4.1***

G × E, gene by environment; GWAS, genome-wide association study; GI, gluten index; DGC, dry gluten content; WGC, wet gluten content; RGC, residual gluten content; WHC, water-holding capacity; ^⁎⁎⁎^Significance at 0.1 % level.

### 3.2 Population structure and LD analysis

**Fig. 2:**
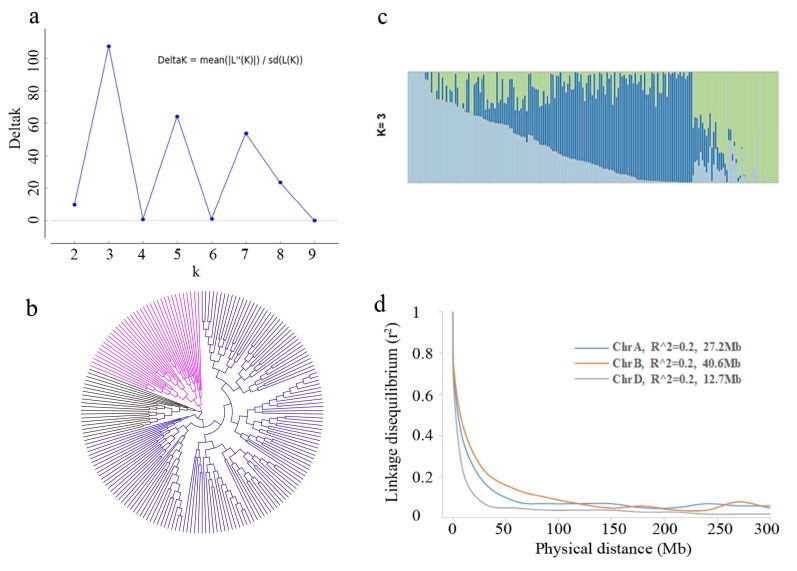
Analysis of the population structure and linkage disequilibrium of the 200 wheat accessions. a, Plot of delta K against putative K values obtained from Structure Harvester analysis. b, Archaeopteryx tree of 200 wheat accessions. c, Population structure for the best subpopulations values. d LD decay of the three sub-chromosomes.

The population structure of 200 AMP wheat genotypes was analyzed using both ΔK and neighbor-joining approaches. The ∆K value peaked at K = 3 but was remained high for K = 5 and K = 7, indicating that K = 3 was the most likely forecast for the number of subpopulations (SPs) (Fig. 2a, c). A similar result was obtained from the neighbor-joining tree analysis (Fig. 2b). In general, Subgroup I, contained 37 accessions, primarily from Henan and Shandong provinces; Subgroup II consisted of 20 accessions, primarily comprising varieties from Sichuan and Hubei provinces; and Subgroup III contained 141 accessions, the majority of which were from Henan, Hebei and Shaanxi provinces, as well as from abroad (Table S1).

**Fig. 3:**
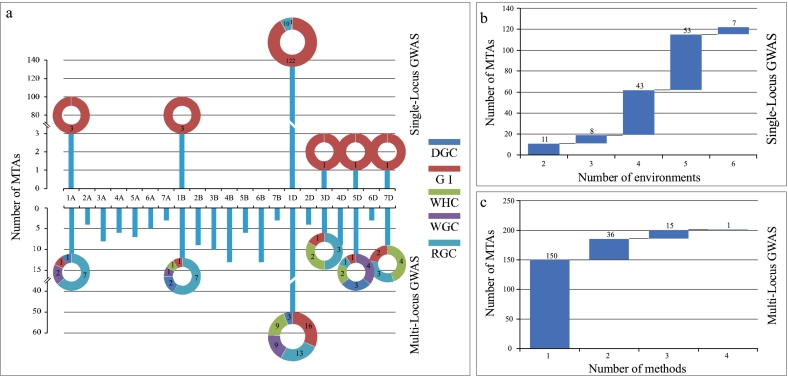
Distribution and statistics of MTAs for gluten-related traits in the AMP using two GWAS models. a, The histogram showed the chromosomal distribution of the discovered MTAs; the sunburst chart showed the distribution of MTAs on specific chromosomes for each parameters; The colors and numbers on the sunburst chart represented different gluten characteristics and MTA quantities, respectively. b, The waterfall plot displays the statistics of detected MTAs in various environments. c, The waterfall plot revealed the statistics of MTAs found using various GWAS methodologies. DGC, dry gluten content; GI, gluten index; WHC, water-holding capacity; WGC, wet gluten content; RGC, residual gluten content.

The level of LD decay for the A, B, and D subgenomes was assessed by calculating the squared correlation coefficient (r^2^) against physical distance. The mean r^2^ values for these three sub-genomes progressively decreased as pairwise distance increased (Fig. 3d). The LD dropped to the threshold r^2^ value (0.2) was estimated for the A, B, and D sub-genomes at approximately 27.2 Mb, 40.6 Mb, and 12.7 Mb, respectively. These values were utilized as the confidence intervals of each sub-genome to find QTLs. To identify QTLs, these values were used as the confidence intervals for each subgenome.

### 3.3 GWAS analysis of wheat gluten-related traits

#### 3.3.1 Single-locus GWAS (SL-MLM)

A total of 143 significant MTAs for wheat gluten-related traits were found using the SL-MLM approach across six environments and BLUE values, based on the threshold value of *P* < 1.0E-6 (i.e. -log_10_(*P*) > 5.98). These detected MTAs were distributed on chromosomes 1A, 1B, 1D, 3D, 5D, and 7D, and individually explained 15.27 %–38.50 % of the phenotypic variance (R^2^) (Table S3, Fig. 3a). Of them, 7, 53, 44, 8 and 11 MTAs were repeatedly detected in six, five, four, three and two different environments, respectively (Fig. 3b).

For GI, up to 132 significant MTAs were discovered on chromosomes 1A, 1B, 1D, 3D, 5D, and 7D, with R^2^ values ranging from 15.27 % to 38.50 % (Table S3). Of these, 123 MTAs were found in more than one environment. The majority of MTAs were discovered on chromosome 1D (123), followed by 1A (3) and 1B (3). These MTAs were divided into three robust QTLs based on their physical positions in the Chinese Spring database (IWGSC V1.0, http://www.wheatgenome.org/). On chromosomes 1A and 1B, three MTAs corresponding to a QTL were mapped at 505,536,405-506,854,746 bp and 551,722,291-553,633,466 bp, respectively. On chromosome1D, 123 MTAs classified as a single QTL were found at 408,346,686-416,456,905 bp. Within this QTL, seven MTAs flanking *AX-109870609*, *AX-95630667*, *AX-108825130*, *AX-110936920*, *AX-109965095*, *AX-111639460*, and *AX-109896332* were stably detected in all six environments and BLUE (Table S3).

For RGC and WGC, 11 significant MTAs were discovered, all of which were on chromosome 1D and detected in only one environment (Table S3). Each MTA explained 16.00–21.62 % of the phenotypic variance. Ten of them, for RGC, were found at 410,464,657-414,587,809 bp and belonged to a single QTL. At 416,359,964 bp, there was only one MTA for the WGC (Table S3). There were no significant MTAs for DGC or WHC.

#### 3.3.2 Multi-locus GWAS

Five different multi-locus GWAS models were used to find 203 significant MTAs for wheat gluten-related (*P* < 1.0E-6, −log10(P) > 5.98). These MTAs were distributed across all 21 wheat chromosomes and had an R^2^ value of 2.38–37.61 % (Supplementary Table S4). Of the total MTAs, 151 were identified exclusively by a single method. In contrast, a smaller subset was consistently detected by multiple methods, with 37, 14, and 1 MTA being identified by two, three, and four methods, respectively (Table S4, Fig. 3c).

46 significant MTAs for GI were found on 17 chromosomes, with the exception of 2A, 4A, 6A and 6D. Each discovered MTA accounted for 2.38–37.61 % of the phenotypic variance (Table S4). Twelve of these 46 MTAs were detected by more than one multi-locus GWAS model. Of which, two MTAs surrounding *AX-111715861* and *AX-111216618* on chromosomes 1D and 5B, respectively, were identified seven times across three environments using three different methods. On chromosome 6B, one MTA flanking *AX-111784251* was discovered four times using three different approaches. One robust QTL that was integrated from 15 significant MTAs at 411,085,238-416,362,032 bp of chromosome 1D was categorized based on physical locations (Table S4).

With R^2^ values ranging from 5.40 % to 10.85 %, a total of 17 MTAs for DGC were found on chromosomes 1A, 1B, 1D, 2A, 2B, 4A, 4B, 5A, 5B, and 5D (Table S4). Out of them, five MTAs were consistently identified using multiple models. On chromosome 4A, one MTA flanking *AX-110008463* was found four times using three different GWAS methods.

Up to 38 major MTAs for WGC were discovered on chromosomes 1A, 1B, 1D, 2A, 3A, 3B, 4A, 4B, 4D, 5D, 6A, 6B, and 6D. These MTAs accounted for 4.16–16.75 % of the variation in phenotype (Table S4). Of these 38 MTAs, eleven could be consistently detected using several models. Each of the two MTAs flanking *AX-111650990* and *AX-109438852* on chromosomes 2A and 4B was found three times by three methods, whereas the one MTA flanking *AX-110087433* on chromosome 5D was found four times by three methods. At 413,295,707-415,766,931 bp of chromosome 1D, five MTAs were grouped together to form a robust QTL (Table S4).

Except for 2A, 4A, and 6D, 66 significant RGC MTAs were identified on 18 chromosomes. The R^2^ values for each MTA ranged from 3.80 % to 22.83 % (Table S4). Sixteen of the 66 MTAs were found frequently using several ML-GWAS methods. One MTA flanking *AX-94666112* on chromosome 1B was discovered four times using three methods, whereas four MTAs flanking *AX-108824021*, *AX-109348990*, *AX-110353768*, and *AX-109841712* on chromosomes 1A, 1D, 1D, and 4B were detected three times. Thirteen MTAs clustered around a robust QTL at 408,245,120-416,362,032 bp on chromosome 1D, while four clustered around a robust QTL at 510,850,578-511,599,359 bp on chromosome 1A (Table S4).

Up to 36 significant MTAs for WHC were discovered on chromosomes 1B, 1D, 2A, 2B, 2D, 3B, 3D, 4A, 4B, 5D, 6A, 6B, 6D, 7B, and 7D, with R^2^ values ranging from 4.38 to 23.27 % (Table S4). Eight of the 36 MTAs were found using more than one ML-GWAS models. One MTA flanking *AX-111988052* on chromosome 1D was detected four times using four methods, whereas one MTA flanking *AX-110087433* on chromosome 5D was discovered three times using three methods. One strong QTL integrated from eight MTAs was found on chromosome 1D at 415,413371-416,094,414 bp (Table S4).

#### 3.3.3 Reliable QTLs for gluten-related traits

In this investigation, a QTL was deemed reliable if it was found more than once, in many environments, and by multiple GWAS models. A total of 15 reliable QTLs were found on chromosomes 1A, 1B, 1D, 2B, 3D, 4A, 4D, 5A, 5B, 5D, 6B, 7D, and 3.12–38.50 % of the phenotypic variation, respectively (Table 2). The number of reliable QTLs for GI, WGC, DGC, RGC and WHC were nine, two, one, three and tree, respectively. Four QTLs on chromosome 1D, *qGI·1D*, *qWGC·1D*, *qRGC·1D*, and *qWHC·1D*, shared the common marker intervals at 408.25–416.46 Mb. These QTLs were found more than eight times and in more than four environments. In particular, *qGI·1D* for GI, which integrated from 138 significant MTAs, was discovered 651 times and recognized by all six ML-GWAS models across all environments. *QWGC·5D* for WGC and *qWHC·5D* for WHC were co-located on chromosome 5D at 544.18–545.52, respectively, and were detected by three distinct models in three distinct environments. *qGI.1A* for GI and *qRGC.1A* for RGC were found to be on chromosome 1A in nearby regions, and were detected in five and three environments by more than two GWAS approaches, respectively. Furthermore, *qGI.1A*, *qGI·1D*, *qGI·7D*, *qWGC·1D*, and *qRGC·1D* were discovered concurrently by SL-MLM and ML GWAS models.

**Table 2:** Stable QTLs for gluten-related traits.

Traits	QTL	Markers	Chr.	No. of MTAs	Position (Mb)	-log_10_(P)	R^2^ (%)	Env.	Method	Detected times
GI	*qGI.1A*	*AX-108958676*	1A	4	505.54–510.85	6.02–10.82	7.56–29.90	E1/E2/E3/E6/BLUE	5,6	10
	*qGI·1B*	*AX-111503025*	1B	3	551.72–553.63	5.99–9.56	18.15–30.61	E1/E2/E3/E6/BLUE	6	15
	*qGI·1D*	*AX-109913657*	1D	138	408.35–416.46	5.99–31.86	15.54–38.50	E1/E2/E3/E4/E5/E6/BLUE	1,2,3,4,5,6	651
	*qGI·4D*	*AX-89640014*	4D	2	113.32–116.82	6.10–7.61	3.12–10.03	E2/E3/BLUE	1,2,3,5	5
	*qGI·5B*	*AX-111216618*	5B	1	549.85	6.67–9.78	5.23–10.14	E2/E4/BLUE	1,2,5	7
	*qGI·6B*	*AX-110940435*	6B	3	45.52–55.24	6.27–13.19	5.60–11.55	E1/E2/E3/E4/E5/BLUE	1,2,5	7
	*qGI·7D*	*AX-109293862*	7D	2	321.05	6.01–12.86	20.40–30.71	E1/E2/E3/E4/E6/BLUE	1,6	7
WGC	*qWGC·1D*	*AX-111888532*	1D	6	413.30–416.36	6.16–12.33	9.17–15.90	E2/E3/E5/BLUE	1,2,4,6	8
	*qWGC·5D*	*AX-110087433*	5D	2	544.18–545.52	6.80–11.17	7.33–13.98	E2/E3/BLUE	1,2,5	5
DGC	*qDGC.4A*	*AX-110008463*	4A	1	624.31	6.31–10.52	11.26–12.44	E2/E3/BLUE	1,2,5	4
RGC	*qRGC.1A*	*AX-110370624*	1A	5	510.85–521.30	6.04–14.30	5.17–14.47	E1/E4/BLUE	1,2,3,4	7
	*qRGC·1D*	*AX-109913657*	1D	23	408.25–416.36	6.03–18.28	4.30–22.83	E2/E3/E4/E6/BLUE	1,2,3,4,5,6	29
	*qRGC·2B*	*AX-109337411*	2B	2	10.79–13.77	6.17–8.61	3.93–5.10	E5/E6/BLUE	1,2,5	6
WHC	*qWHC·1D*	*AX-111988052*	1D	7	415.41–416.09	6.97–24.06	10.62–23.27	E2/E3/E5/BLUE	1,2,3,4,5	11
	*qWHC·5D*	*AX-110087433*	5D	2	544.48–545.52	7.89–9.72	6.39–11.10	E2/E3/BLUE	2,3,5	4

GI, gluten index; WGC, wet gluten content; DGC, dry gluten content; RGC, residual gluten content; WHC, water-holding capacity; Marker: representative markers at the corresponding QTL; Chr., chromosome; MTAs, marker-trait associations; Position, the location in the entire genome database of Chinese Spring; R^2^, phenotypic variance explained by the SNPs; E1, 2014–2015 in Huixian, E2, 2016–2017 in Huixian; E3, 2017–2018 in Huixian; E4, 2018–2019 in Huixian; E5, 2017–2018 in Xinxiang; E6, 2018–2019 in Xinxiang; BLUE, the best linear unbiased estimator values based on all environments; 1, mrMLM; 2, FASTmrMLM; 3, FASTmrEMMA; 4, pLARmEB; 5, pKWmEB; 6, SL-MLM.

#### 3.3.4 Candidate gene analysis of gluten-related traits

The aforementioned 15 trustworthy QTLs were chosen as future study targets because they were consistently expressed in diverse environments or recognized by several GWAS methods. The interval sequences of significant SNP markers were used to identify wheat gluten candidate genes. In the early stage, as shown in Table 3, ten potential wheat gluten genes were found. Four candidate genes were discovered in *qGI.1A*, three in *qGI·1B*, and the remaining three in two QTLs (*qGI·1D* and *qRGC·1D*).

**Table 3:** Putative Candidate Genes of wheat gluten parameters.

Chr	QTL	Border markers	Candidate genes	Physical position (bp)	Function description
1A	*qGI.1A*	*AX-108958676*	*TraesCS1A01G466100LC*	508,724,002-508,726,319	HMW-GS
			*TraesCS1A02G466400LC*	508,914,854-508,924,980	HMW-GS
			*TraesCS1A02G466500LC*	508,924,985-508,925,603	HMW-GS
			*TraesCS1A02G317500*	508,929,435-508,930,124	HMW-GS
1B	*qGI·1B*	*AX-111503025* *AX-108999948* *AX-94670671*	*TraesCS1B01G569900LC*	555,845,968-555,848,608	HMW-GS
			*TraesCS1B01G570600LC*	555,933,438-555,935,808	HMW-GS
			*TraesCS1B02G330000*	555,938,670-555,939,829	HMW-GS
1D	*qGI·1D* *qRGC·1D*	*AX-109913657* *AX-110353768*	*TraesCS1D01G435600LC*	412,160,789–412,163,311	HMW-GS
			*TraesCS1D01G435700LC*	412,217,724–412,219,723	HMW-GS
			*TraesCS1D02G317300*	412,223,432–412,224,112	HMW-GS

Chr, chromosome; HMW-GS, high molecular weight glutenin subunit.

**Fig. 4:**
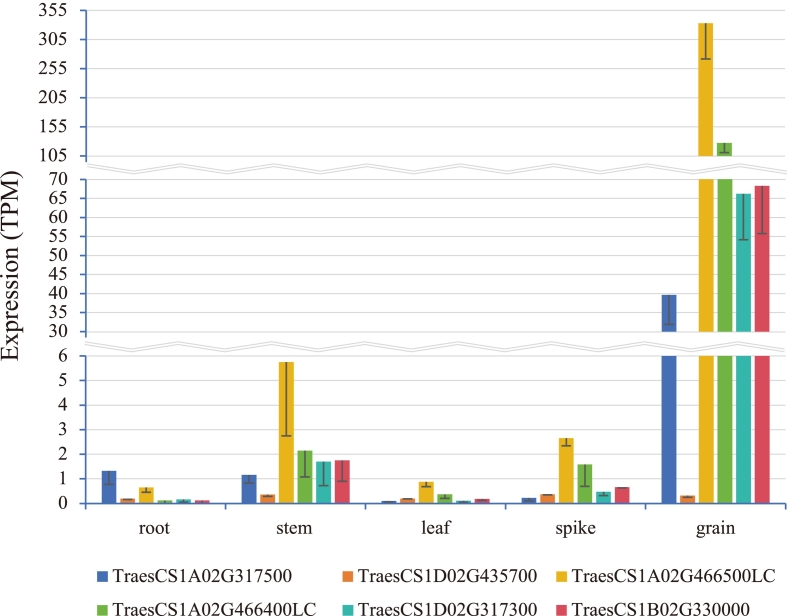
Expression analysis (TPM, transcripts per million)of candidate genes in five wheat tissues.

To narrow down the potential gene range, gene expression in the Chinese Spring Development wheat expression database (single) was searched via the WheatOmics website. Five of the ten genes (*TraesCS1A02G317500*, *TraesCS1A02G466400LC*, *TraesCS1A02G466500LC*, *TraesCS1B02G330000* and *TraesCS1D02G317300*) were found to be expressed to varied degrees in five wheat tissues (root, stem, leaf, spike, and grain) (Fig. 4). And it was found that these five genes were highly expressed in grain tissue. Given that wheat seeds are an essential storage place for gluten protein, we might conclude that these five genes are candidates for influencing gluten-related features in this research population.

**Fig. 5:**
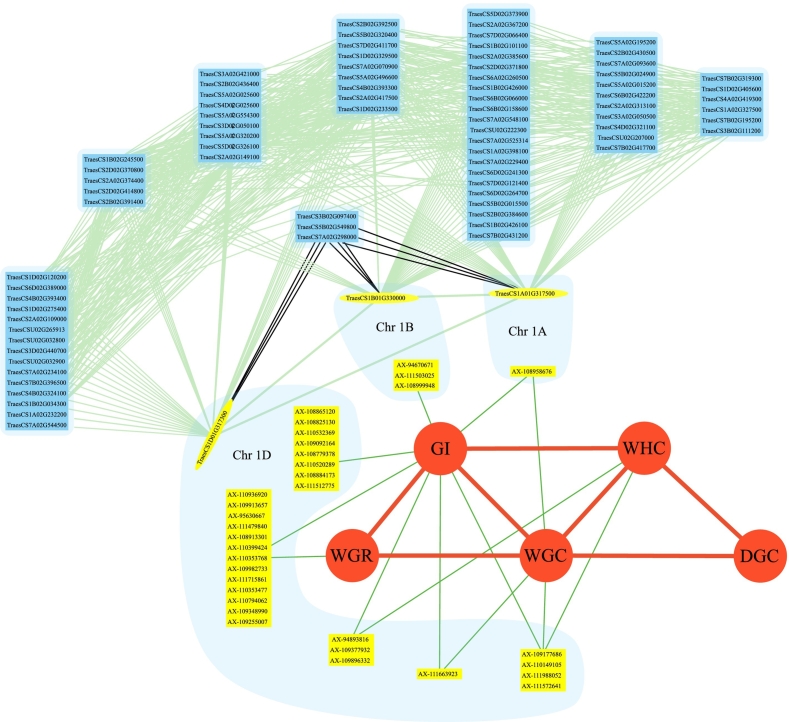
Network analysis for gluten parameters, MTAs and candidate genes.

To better understand the relationship between phenotypes and genes, we also performed network analysis of phenotypes and candidate genes (Fig. 5). The wheat gluten genotype-phenotype network showed that five gluten characteristics correlated differently. GI was directly associated to the other three indicators (except DGC), while DGC was indirectly related to GI via the other two indicators (WGC and WHC). There was a direct relationship between WGR and GI, as well as WGR and WGC. A network diagram for the five potential genes described was created by scanning the *T.aestivum* (Chinese spring)_AABBDD global network on the WheatCENet website. The developed gene network map contained just three candidate genes (*TraesCS1A02G317500*, *TraesCS1B02G330000*, and *TraesCS1D02G317300*), and their expression levels were positively correlated. Furthermore, they showed a negative correlation with the expression of three other genes (*TraesCS3B02G097400*, *TraesCS5B02G549800*, and *TraesCS7A02G298000*).

Traits and MTAs that were closely related to candidate genes were represented by red dots and yellow boxes. Genes were represented by blue boxes. Shadows surround candidate genes and MTAs that were closely related with them. Red, green and light green lines reflect correlations between traits, traits and MTAs, and genes. Black lines showed a negative gene expression association.

### 3.4 Establishment and verification of KASP markers

**Fig. 6:**
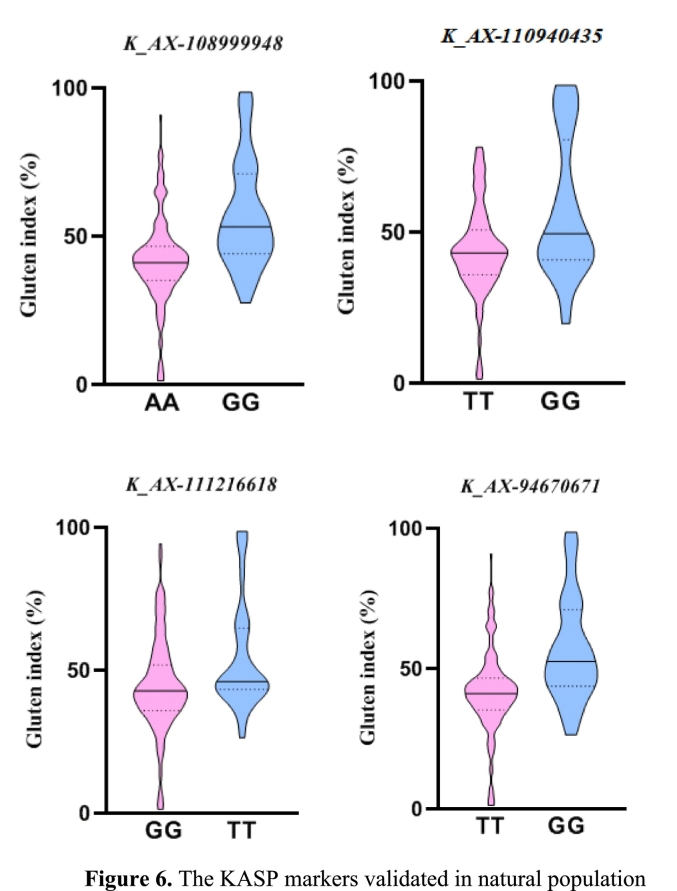
The KASP markers validated in natural population.

While conventional breeding has played a key role in wheat quality enhancement, its reliance on challenging field measurements renders the selection process slow and inefficient (Rasheed et al., 2016). The KASP technology addresses these limitations by enabling a cost-effective, flexible, and precise strategy for MAS. In this work, we developed four high-fidelity KASP markers (*K_AX-108*,*999*,*948*, *K_AX-110*,*940*,*435*, *K_AX-111*,*216*,*618*, *K_AX-94*,*670*,*671*) for GI from tightly linked SNPs; validation in breeding programs confirmed their practical utility. Genotyping consistency with the SNP platform was above 0.95 for all markers. As shown in Table 4, For *K_AX-108*,*999*,*948*, the favorable allele (GG), carried by 25.4 % of individuals, was associated with a significantly higher mean GI of 57.2 % compared to the unfavorable allele (AA) (74.6 %, mean GI, 41.5 %) (*p* < 0.05). Analysis of *K_AX-110*,*940*,*435* revealed that the favorable allele (GG) (15.1 %) correlated with a higher mean GI (57.2 %), while the unfavorable allele (TT) (84.9 %) was linked to a lower mean GI of 43.6 % (p < 0.05). For *K_AX-111*,*216*,*618*, although less frequent (13.9 %), the favorable allele (TT) was associated with a significantly higher mean GI of 54.5 % compared to the predominant unfavorable allele (GG) (86.1 %, mean GI, 44.3 %) (p < 0.05). For *K_AX-94*,*670*,*671*, the favorable allele (GG) account for 26 % (mean GI: 56.6 %) exhibited higher GI compared to the unfavorable allele (TT), which account for 74 % with mean GI 41.5 % (p < 0.05) (Fig. 6). Promising accessions, including Xiaobingmai 33 and Fengyou 7, which combine multiple favorable alleles with excellent grain quality and agronomic traits, are therefore identified as recommended parents for wheat quality breeding.

**Table 4:** The KASP markers validated in the diverse panel.

Marker	QTL	Genotype	Number of lines	Phenotype (%)	*P* value
*K_AX-108*,*999*,*948*	*qGI·1B*	AA	144	GI: 41.5	*P* < 0.05
		GG	49	GI: 57.2	
*K_AX-110*,*940*,*435*	*qGI·6B*	TT	163	GI: 43.6	*P* < 0.05
		GG	29	GI: 57.2	
*K_AX-111*,*216*,*618*	*qGI·5B*	GG	167	GI: 44.3	*P* < 0.05
		TT	27	GI: 54.5	
*K_AX-94*,*670*,*671*	*qGI·1B*	TT	142	GI: 41.5	*P* < 0.05
		GG	50	GI: 56.6

## 4 Discussion

### 4.1 Important QTLs for wheat gluten-related traits

A total of 143 and 203 significant MTAs for wheat gluten-related traits were found in this investigation using one SL-GWAS model and five ML-GWAS models, respectively. We evaluated the physical locations of these MTAs by comparing their marker sequences to genome sequences from Chinese Spring (IWGSC V1.0, http://www.wheatgenome.org/). Eventually, up to 15 credible QTLs were found on chromosomes 1A, 1D, 2B, 4A, 4D, 5B, 5D, 6B and 7D; they were discovered more than four times in at least two environments and by several GWAS models. As a result, they were designated as relevant genetic areas for further investigation. They were therefore identified as trustworthy genetic regions worthy of further investigation.

The high-molecular-weight gluten subunits (HMW-GS), which are an essential part of glutenin and primarily affect the final quality of dough, are encoded by the known genes *Glu-1Ax* (508.723–508.726 Mb) and *Glu-1Ay* (508.924–508.925 Mb) on chromosome 1A, *Glu-1Bx* (555.765–555.766 Mb) and *Glu-1By* (555.933–555.935 Mb) on chromosome 1B, as well as *Glu-1Dx* (412.160–412.163 Mb) and *Glu-1Dy* (412.217–412.219 Mb) on chromosome 1D (Zang et al., 2022). In this study, two credible QTLs (*qGI.1A* and *qRGC.1A*) on chromosome 1A, one QTL (*qGI·1B*) on chromosome 1B and four credible QTLs (*qGI·1D*, *qWGC·1D*, *qRGC·1D* and *qWHC·1D*) on chromosome 1D were mapped in the same or nearby genomic regions as these known genes. Of them, *qGI·1B* was detected 15 times in five environments, but only by one model, whereas the remaining QTLs were all discovered more than seven times in at least three environments by seveal models. At 408.25–416.46.46 Mb of chromosome 1D, *qGI·1D* for GI and *qRGC.1D* for RGC, respectively, were found 651 and 29 times by all GWAS models. These regions contained 138 and 23 significant MTAs and explained 15.54–38.50 % and 4.30–22.83 % of phenotypic variance, respectively. Additionally, QTLs associated to wheat gluten were previously mapped in these same chromosomal areas on chromosomes 1A, 1B, and 1D, respectively (Barakat et al., 2020; Deng et al., 2015; Liu et al., 2017; Yang et al., 2020a). These outcomes confirm the validity of the study's findings, and additional research can be conducted using these trustworthy loci as its main focus.

Two trustworthy QTLs (*qWGC·5D* and *qWHC·5D*) with R^2^ values 7.33–13.98 % and 6.39–11.10 %, respectively, were co-localized at around 544.19–545.52 Mb on chromosome 5D. Three GWAS models consistently found these two QTLs in three distinct environments. A chromosome 5D QTL for WGC, *QWgc.sicau-5DL*, was previously mapped by Pu, et al. at approximately 555.91 Mb (Pu et al., 2022); Li, et al. found one meta-QTL for WGC at 517.97–625.23 Mb (Li, Wen, et al., 2019); Yang, et al. also discovered multiple QTLs for WGC at approximately 495 Mb (Yang et al., 2020b); and Barakat et al. (2020) located one QTL for GI, *GI.hs-5D*, at 491 Mb. Additionally, QTLs regulating wheat protein content were found in this chromosomal region by Hao et al. (2022) and Aoun et al. (2022), suggesting that this genomic region would be crucial for enhancing the quality of wheat and that important loci linked to wheat gluten-related traits might be found on chromosome 5D.

One stably expressed QTL for GI, *qGI·7D*, was mapped at 321.05 Mb on chromosome 7D, explaining 20.40–30.71 % of the phenotypic variance, and was consistently discovered in all test environments except environment E5. Barakat, et al. identified one QTL for GI, *GI.n-7D*, which accounted for 21.0 % of the phenotypic variance within the interval Excalibur_rep_c85891_96- Ra_c1530_372 (237.5–446.6 Mb) of chromosome 7D (Barakat et al., 2020). Further research may be required to confirm whether these two QTLs influencing GI are identical. *qRGC·2B* for RGC was found on chromosome 2B at 10.79–13.77 Mb, and three GWAS models consistently identified it in three different environments. A core QTN region linked to WGC was earlier reported at 5.67–9.89 Mb of chromosome 2B (Yang et al., 2020a). This region was also linked to total starch content, dough water absorption, SDS-sedimentation volume (SV), and grain protein content (GPC). Additionally, a few QTLs for SV and GPC were previously discovered by certain researchers in nearby genomic regions (Pu et al., 2022; Ruan et al., 2020; Suliman et al., 2021). These findings imply that significant loci governing wheat gluten-related parameters as well as other quality traits might be found on chromosome 2B. Additional research is required to determine whether the loci granting these traits are contiguous to one another or identical. However, the remaining three QTLs (*qGI·4D*, *qGI·5B*, and *qDGC.4A*) on chromosomes 4D, 5B, and 4A, respectively, did not overlap with or deviate from the previously identified QTLs for wheat gluten-related traits, suggesting that they might represent new QTLs for these traits.

### 4.2 Potential candidate genes for wheat gluten parameters

All ten potential genes identified in the early stages of this investigation were found on the *Glu-1* loci (*Glu-A1*, *Glu-B1* and *Glu-D1*), concentrated around significant markers and within the confidence intervals of LD decay values for each subgenome. Their functional annotations and domains were labeled as HMW-GS. By searching the expression status of candidate genes through the Chinese Spring Wheat Expression Database, it was found that five genes (*TraesCS1A02G317500*, *TraesCS1A02G466400LC*, *TraesCS1A02G466500LC*, *TraesCS1B02G330000*, and *TraesCS1D02G317300*) are overexpressed in wheat grain tissues (Mayer et al., 2014). *TraesCS1A02G317500* encodes a protein that is speculated to have a function on Bifunctional inhibitor, plant lipid transfer protein, or seed storage helical domain. The percentage of identical matches with *TraesCS1B02G330000* and *TraesCS1D02G317300* have reached 96.5 % and 91.2 % respectively, indicating that the genes with this sequence may play an important role in wheat gluten parameters. The gene *TraesCS1B02G330000* has the PF13016 domain suggesting a relationship with gliadins. *TraesCS1A02G466400LC and TraesCS1A02G466500LC* encoding HMW gluten and having a function on nutrient reservoir activity, are highly expressed in the grain, indicating the two genes affecting gluten parameters directly.

Gong et al. found that Tr*aesCS1A02G317500* and *TraesCS1B02G330000* were upregulated in the high-GPC group against the low-GPC group (Gong et al., 2022). Furthermore, Yang, et al. proposed that *TraesCS1B02G330000* directly contributes to the creation of wheat grain quality (Yang et al., 2020b). This suggests that these two genes were critical in the accumulation of wheat grain protein. Gluten, as the most essential component of wheat grain protein, may be strongly linked to these two genes.

Muqaddasi, et al. discovered that the gene *TraesCS1D02G317300* was linked to a major effect QTL (*QZsv.ipk-1D*) that controlled wheat Zeleny sedimentation value, which is directly related to gluten characteristics (Muqaddasi et al., 2023enes (*TraesCS1A02G317500*, *TraesCS1B02G330000*, and *TraesCS1D02G317300*) and their neighboring genes exhibited high collinearity (Fig. S1). Because these three genes are substantially expressed in grains and have a high degree of collinearity, it is possible that they are also conserved in gene function. In other words, these homologous genes may perform similar biological processes and play a critical role in grain development and quality generation.

### 4.3 Potential regulatory networks for wheat gluten parameters

Wheat gluten parameters, as quantitative traits, are formed by a complex biological process that requires the cooperation of many genes. Five candidate genes (*TraesCS1A02G317500*, *TraesCS1A02G466400LC*, *TraesCS1A02G466500LC*, *TraesCS1B02G330000* and *TraesCS1D02G317300*) express themselves particularly in wheat grain tissues and encode HMW-GS. Although the concentration of HMW-GS in wheat grain was low, accounting for just approximately 10 % of the typical storage protein, it might explain almost 60 % of the variation in bread baking quality (Li et al., 2023). By network analysis, it was found that three genes (*TraesCS1A02G317500*, *TraesCS1B02G330000* and *TraesCS1D02G317300*) among the five candidate genes had positive correlation with most of the other genes and adversely linked with the other three genes (*TraesCS3B02G097400*, *TraesCS5B02G549800*, and *TraesCS7A02G298000*) (Fig. 5), confirming their complex correlation in spatiotemporal expression.

*TraesCS3B02G097400* encodes a trichome birefringence-like protein that is linked to cell wall polysaccharide *O*-acetylation in Arabidopsis (Schultink et al., 2015), cell wall construction in quinoa (Ma et al., 2021), fiber development in upland cotton (Yu & Gervers, 2019), and powdery mildew resistance in *Lupinus perennis* (Wilson, 2019). *TraesCS5B02G549800* encodes RPM1-interacting protein 4 (RIN4), a conserved plant immune regulator that can be affected by pathogenic effector proteins (Zhao et al., 2021). RIN4 is required for RPM1-mediated disease resistance because it promotes the accumulation of RPM1 prior to infection (Belkhadir et al., 2004). *TraesCS7A02G298000* is referred to as Chaperone protein dnaJ. Classical dnaJ proteins work as co-chaperones with HSP70 to manage protein homeostasis, and most cellular proteins require chaperone care at various phases of their life (Pulido & Leister, 2018). The DnaJ proteins, also known as heat shock protein 40 according to their molecular weight, respond to heat stress in plant growth (Fan et al., 2017). All three genes play critical roles in environmental stress response. It is possible that the variations in carbon and nitrogen buildup produced by environmental stress during grain filling impact the amount and composition of starch and storage protein, resulting in lower yield and quality attributes.

### 4.4 The future directions in wheat grain quality breeding

Wheat quality breeding is no longer limited to increasing grain protein content, but is developing towards more precise, efficient, and diverse directions. Advances in functional genomic studies of quality-related traits and the development of MAS have greatly accelerated the improvement of end-use quality in wheat. Many genes that contribute to quality-related traits have been utilized in breeding. Gene-specific markers corresponding to various glutenin subunit alleles have been developed for gene tagging and marker-assisted transfer. In this study, we identified QTLs on chromosome 1A, 1B and 1D. Interestingly, the three genes (*TraesCS1A02G317500*, *TraesCS1B02G330000*, and *TraesCS1D02G317300*) exhibited high collinearity, and *TraesCS1B02G330000* has the PF13016 domain suggesting a relationship with gliadins, suggesting that gliadin maybe a crucial index for further wheat grain quality breeding.

High-throughput KASP technology accelerates wheat breeding by enabling rapid screening of elite germplasm in early generations (Rasheed et al., 2016). Although conventional breeding is crucial for enhancing processing quality, combining it with marker-assisted selection (MAS) substantially improves precision and efficiency. Genomic loci that are stable across environments, such as the *qGI·1B* (marked by *K_AX-108*,*999*,*948*) for wheat GI, are ideal candidates for implementation in MAS breeding programs. Additionally, the reliability of several earlier-reported loci has been reinforced through validation, demonstrating their common occurrence in diverse germplasm. These loci are thus prime targets for MAS. Favorable alleles identified in natural populations can be directly introduced into breeding programs via elite parental lines. By leveraging these markers and selected germplasm, breeders can streamline the development of wheat with enhanced processing quality, driving long-term industry improvement.

## 5 Conclusions

Integration of single-locus and multi-locus GWAS models improved our understanding of the genetic architecture of wheat gluten-related traits. This study confirmed the findings of earlier loci linked to gluten-related traits and discovered several novel loci associated with wheat gluten parameters. A noteworthy finding was the identification of 15 reliable QTLs that were detected repeatedly, across a variety of environments, and by several GWAS methods. In addition, five putative genes (*TraesCS1A02G317500*, *TraesCS1A02G466400LC*, *TraesCS1A02G466500LC*, *TraesCS1B02G330000* and *TraesCS1D02G317300*) on chromosome 1A, 1B and 1D were hypothesized to be candidate genes regulating wheat gluten parameters. They exhibit spatiotemporal expression correlation and a negative correlation with three additional genes (*TraesCS3B02G097400*, *TraesCS5B02G549800*, and *TraesCS7A02G298000*) linked to the response to environmental stress. In the process of developing MAS for wheat quality breeding, these findings should prove useful.

## CRediT authorship contribution statement

**Xiaoling Jiang:** Writing – original draft, Visualization, Formal analysis, Conceptualization. **Qiang Li:** Writing – review & editing, Investigation, Formal analysis. **Yanyan Geng:** Investigation, Formal analysis. **Jishun Zhao:** Investigation. **Yang Li:** Investigation. **Hongmin Li:** Writing – review & editing, Writing – original draft, Visualization, Supervision, Project administration, Formal analysis, Conceptualization.

## Declaration of competing interest

The authors declare that they have no known competing financial interests or personal relationships that could have appeared to influencethe work reported in this paper.

## Data Availability

Data will be made available on request.

## Appendix A Supplementary data

Supplementary material 1 Table S1. List of 200 wheat accessions used for the GWAS analysis. Table S2. Distribution of SNPs on 21 wheat chromosomes. Table S3. SNPs associated with wheat gluten-related traits based on a single-locus genome-wide analysis in six environments and BLUE. Table S4. SNPs associated with wheat gluten-related traits based on five multi-locus genome-wide studes in six environments and BLUE. Table S5. KASP markers information. Fig. S1. Collinearity analysis of three candidate genes for gluten parameters. Supplementary material 1
